# Supplementary material for: A unified computational framework for single-cell data integration with optimal transport
Source: Nat Commun. 2022 Dec 1;13:7419. doi: 10.1038/s41467-022-35094-8 (PMC9715710; doi:10.1038/s41467-022-35094-8)
Supplement: Supplementary file 1 — Supplementary Information [file 41467_2022_35094_MOESM1_ESM.pdf]

Supplementary Information

uniPort for single-cell data integration

Kai Cao, Qiyu Gong, Yiguang Hong, and Lin Wan

Supplementary Method 1: Minibatch unbalanced optimal transport

Here we give a brief introduction to optimal transport (OT), which has attracted more and more attention in the analysis of single-cell datasets recently. Suppose we have two single-cell datasets $\mathbf{X} = \{\mathbf{x}_i\}_{i=1}^{n_x}, \mathbf{x}_i \in \mathbb{R}^K$ and $\mathbf{Y} = \{\mathbf{y}_j\}_{j=1}^{n_y}, \mathbf{y}_j \in \mathbb{R}^K$ in a K-dimensional spaces. Two discrete measures with weights $\mathbf{a}$, $\mathbf{b}$ are defined as

$$\mathbf{a} = \sum_{i=1}^{n_x} a_i \delta_{\mathbf{x}_i}, \; \mathbf{b} = \sum_{i=1}^{n_y} b_i \delta_{\mathbf{y}_i} \tag{1}$$

where $\delta_{\mathbf{x}}$ is the Dirac at cell $\mathbf{x}$, and $\mathbf{a}$, $\mathbf{b}$ belong to the probability simplex: $\Sigma_n \stackrel{\text{def}}{=} \{\mathbf{a} \in \mathbb{R}_+^n : \sum_{i=1}^n \mathbf{a}_i = 1\}$. Typically, we initialize $\mathbf{a}$, $\mathbf{b}$ as uniform distribution with each element be the same, that is $\mathbf{a} = \frac{1}{n_x}\mathbf{1}_{n_x}, \mathbf{b} = \frac{1}{n_y}\mathbf{1}_{n_y}$. However, we also provide a user-guided reweight option in our method if the importance of each cell for alignment is not the same.

The OT plan, i.e., cell-cell probabilistic coupling matrix, $\mathbf{T} \in \mathbb{R}_+^{n_x \times n_y}$, as well as a cost matrix $\mathbf{C} \in \mathbb{R}_+^{n_x \times n_y}$, describes the probabilities of aligning cells and the distances between cells across datasets, respectively. Consequently, the discrete OT problem is defined as follows

$$\boldsymbol{L}(\mathbf{a}, \mathbf{b}) \stackrel{\text{def}}{=} \min_{\mathbf{T} \in \Pi(\mathbf{a}, \mathbf{b})} \langle \mathbf{C}, \mathbf{T} \rangle = \min_{\mathbf{T} \in \Pi(\mathbf{a}, \mathbf{b})} \sum_{i,j} \mathbf{C}_{ij} \mathbf{T}_{ij} \tag{2}$$

where $\Pi(\mathbf{a}, \mathbf{b}) \stackrel{\text{def}}{=} \{\mathbf{T} \in \mathbb{R}_+^{n_x \times n_y} : \mathbf{T}\mathbf{1}_{n_y} = \mathbf{a},\; \mathbf{T}^\top \mathbf{1}_{n_x} = \mathbf{b}\}$, and $\langle \cdot, \cdot \rangle$ denotes Frobenius dot product. Besides, in order to introduce some smoothness to the transport matrix, the approximate solution

according to the regularization parameter ϵ writes as

$$\boldsymbol{L}_\epsilon(\mathbf{a},\mathbf{b}) \stackrel{\text{def}}{=} \min_{\mathbf{T}\in\Pi(\boldsymbol{a},\boldsymbol{b})} \langle \mathbf{C}, \mathbf{T}\rangle - \epsilon \sum_{i,j} \mathbf{T}_{i,j}\log(\mathbf{T}_{i,j}) \tag{3}$$

Equation (3) is strictly convex optimization problem and can be solved efficiently via iterative Bergman projections [1]:

$$\boldsymbol{\alpha}^{(l+1)} = \frac{\mathbf{a}}{\mathbf{G}\boldsymbol{\beta}^{(l)}}, \quad \boldsymbol{\beta}^{(l+1)} = \frac{\mathbf{b}}{\mathbf{G}^\top\boldsymbol{\alpha}^{(l)}} \tag{4}$$

starting from $\boldsymbol{\beta}^{(0)} = \frac{1}{n_y}\mathbf{1}_{n_y}$, where $\mathbf{G}_{ij} = e^{-\mathbf{C}_{ij}/\epsilon}$, and the optimal transport plan $\mathbf{T}^*_{ij} = \alpha_i\mathbf{G}_{ij}\beta_j$. In uniPort, we employ a more robust and efficient inexact proximal point method (IPOT) [9] to compute the OT plan. Concretely, uniPort replaces $\mathbf{G}_{ij}$ with $\mathbf{G}'_{ij} = \mathbf{T}^{(l)}_{ij}e^{-\mathbf{C}_{ij}/\epsilon}$ in Eq. (4).

To combine OT with coupled-VAE, we utilize the minibatch unbalanced optimal transport (Minibatch-UOT) [4], which is a geometrically robust version. Minibatch-UOT is computed between batches which is practical for large-scale datasets and deep learning applications, and also decreases the influence of undesired outliers which makes it suitable for partially-overlap datasets. Compared to classic OT, Minibatch-UOT changes the Eq. (3) as

$$\min_{\mathbf{T}\in\mathbb{R}_+^{B_x\times B_y}} < \mathbf{C},\ \mathbf{T} > -\epsilon H(\mathbf{T}) + \rho\left(D_{KL}(\mathbf{T}\mathbf{1}_B\|\mathbf{a}) + D_{KL}(\mathbf{T}^\top\mathbf{1}_B\|\mathbf{b})\right) \tag{5}$$

where B_x and B_y are minibatch sizes of datasets $\mathbf{X}$ and $\mathbf{Y}$, and $\mathbf{T}$ and $\mathbf{C}$ are minibatch OT plan and cost. D_{KL} is KL divergence, and ρ is a marginal penalization. It should be noted that when $\tau \to \infty$, the algorithm degenerates into balanced OT. Therefore, the corresponding Eq. 4 can be rewritten as

$$\boldsymbol{\alpha}^{(l+1)} = \left(\frac{\mathbf{a}}{\mathbf{G}\boldsymbol{\beta}^{(l)}}\right)^{\frac{\rho}{\rho+\epsilon}}, \quad \boldsymbol{\beta}^{(l+1)} = \left(\frac{\mathbf{b}}{\mathbf{G}^\top\boldsymbol{\alpha}^{(l)}}\right)^{\frac{\rho}{\rho+\epsilon}} \tag{6}$$

Besides, uniPort computes the OT cost $\mathbf{C}$ between the Gaussian mixture models $\{\mathcal{N}(\boldsymbol{\mu}_{x_k}, \boldsymbol{\sigma}^2_{x_k}\mathbf{I})\}_{k=1}^{B_x}$ and $\{\mathcal{N}(\boldsymbol{\mu}_{y_k}, \boldsymbol{\sigma}^2_{y_k}\mathbf{I})\}_{k=1}^{B_y}$, instead of latent vectors $\mathbf{z}_x$ and $\mathbf{z}_y$, where $\boldsymbol{\mu}$ and $\boldsymbol{\sigma}$ are output of the probabilistic encoder of coupled-VAE.

Definition 1 *For two K-dimensional Gaussian distributions $\mathcal{N}(\boldsymbol{\mu}_x, \boldsymbol{\Sigma}_x)$ and $N(\boldsymbol{\mu}_y, \boldsymbol{\Sigma}_y)$. The*

Wasserstein distance admits a closed-form expression [8]:

$$W(p_x, p_y) = \|\boldsymbol{\mu}_x - \boldsymbol{\mu}_y\|^2 + trace\left(\boldsymbol{\Sigma}_x + \boldsymbol{\Sigma}_y - 2(\boldsymbol{\Sigma}_x^{\frac{1}{2}}\boldsymbol{\Sigma}_y\boldsymbol{\Sigma}_x^{\frac{1}{2}})^{\frac{1}{2}}\right) \tag{7}$$

When the covariance matrices are diagonal, i.e., $\boldsymbol{\Sigma} = diag(\boldsymbol{\sigma}^2)$*, where* $\boldsymbol{\sigma} \in \mathbb{R}_+^K$ *is the standard deviation vectors, we can rewritten Wasserstein distance as [10]:*

$$W(p_x, p_y) = \|\boldsymbol{\mu}_x - \boldsymbol{\mu}_y\|^2 + \|\boldsymbol{\sigma}_x - \boldsymbol{\sigma}_y\|^2 \tag{8}$$

Therefore, according to Definition 1, the optimal transport cost $\mathbf{C}_{ij}$ between i-th component in $\{\mathcal{N}(\boldsymbol{\mu}_{x_k}, \boldsymbol{\sigma}_{x_k}^2\mathbf{I})\}_{k=1}^{B_x}$ and j-th component in $\{\mathcal{N}(\boldsymbol{\mu}_{y_k}, \boldsymbol{\sigma}_{y_k}^2\mathbf{I})\}_{k=1}^{B_y}$ is defined as

$$\mathbf{C}_{ij} = \|\boldsymbol{\mu}_{x_i} - \boldsymbol{\mu}_{y_j}\|^2 + \|\boldsymbol{\sigma}_{x_i} - \boldsymbol{\sigma}_{y_j}\|^2 \tag{9}$$

We provide a Python package for the implementation of uniPort at `https://github.com/caokai1073/uniPort`. Parts of the code are based on modifications of SCALEX (`https://github.com/jsxlei/SCALEX`) and RAE (`https://github.com/HongtengXu/Relational-AutoEncoders`).

Supplementary Method 2: Global OT plan for high-plex RNA imaging-based and barcoding-based ST data

uniPort can output a global OT plan, i.e., cell-to-spot probabilistic matching matrix, that transfers labels for deconvolution of spatial heterogeneous data across wide-ranging resolutions, such as 10X Visium and microarray-based ST data. After obtaining the OT plan, we can directly use it for deconvolution of mixed spots by summing the transport mass, i.e., probability, of every cluster according to given labels in scRNA data for each spot, which can be written as a matrix $\mathbf{M}$ (row for spot and column for cell cluster). Then we divide each column of $\mathbf{M}$ by the cell number of the corresponding cluster. Users can also use a priori-biased cluster proportion by changing the dividend. $\mathbf{M}$ can also be filtered by top percent clusters according to the user's requirement.

Supplementary Method 3: Contrastive learning with reference guided prior information

To improve the performance of integration, we also develop a contrastive learning [5] method to incorporate cell type annotations or any cell-cell correspondence if available, which was introduced in our former work, Pamona [2], and also proposed by MAT^2 [11]. Following the definition in MAT^2, for cell x_i in dataset $\mathbf{X}$ and cell y_j in reference dataset $\mathbf{Y}$, if they have the same cell-type annotation or a prior correspondence, we regard the cell triplets as a positive anchor, and negative conversely. We combine cell triplets with Minibatch-UOT, which is like the Joint Distribution Optimal Transport (JDOT) [3]. Specifically, we define a prior matrix $\mathbf{F} \in \mathbb{R}^{B_x \times B_y}$ where

$$\mathbf{F}_{ij} = \begin{cases} \alpha, & \text{if triplet } (i,j) \text{ is negative} \\ 1, & \text{if triplet } (i,j) \text{ is unknown} \\ 1/\alpha, & \text{if triplet } (i,j) \text{ is positive.} \end{cases} \tag{10}$$

Here $\alpha \geq 1$ is a user-guided parameter reflecting the confidence of cell-type annotations or correspondence, and larger α means better confidence. We multiply $\mathbf{F}$ with $\mathbf{C}$ to formulate the final transport cost

$$\mathbf{C}_{ij} \leftarrow \mathbf{C}_{ij} * \mathbf{F}_{ij} \tag{11}$$

We added contrastive learning to uniPort for integrating datasets without common features and applied it to integrate PBMC scRNA genes and scATAC peaks data (Supplementary Fig. 16c). Results showed that the guided information of cell-type annotations improved the performance of uniPort for this task, with most of the cell types aligned well.

Supplementary Method 4: Sample reweight during integration

Most single-cell data integrative methods give cells the same importance during integration. However, in some real-world tasks, rare cells in one modality deserve much attention and should be matched with massive cells in other modalities. Therefore, we provide an option for user-guided sample weights if cells should not uniformly matched. In this case, we set **a** and **b** in Minibatch

UOT as weighted distributions **p** and **q** specified by users instead of uniform vectors and reweight reconstruction loss in coupled-VAE as well. Specifically, if users want to give samples in dataset **X** a new weight distribution **p** and samples in dataset **Y** a new **q**, then the Minibatch-UOT loss becomes

$$\min_{\mathbf{T}\in\mathbb{R}_{+}^{B_x\times B_y}} <\mathbf{C},\ \mathbf{T}> -\epsilon H(\mathbf{T}) + \rho\left(D_{KL}(\mathbf{T}\mathbf{1}_B\|\mathbf{p}) + D_{KL}(\mathbf{T}^{\top}\mathbf{1}_B\|\mathbf{q})\right) \tag{12}$$

Supplementary Discussion 1: uniPort imputes genes in an online manner

To explore uniPort's ability for online imputation, we utilized the well-trained model of scRNA and MERFISH data profiled from mouse #1 as a reference atlas and downloaded MERFISH data profiled from mouse #2 with 59,651 cells and 155 genes. We selected the same 153 common genes as in the above-referenced MERFISH data of mouse #2 and input them into the atlas. Then the encoder projected MERFISH data of mouse #2 into the integrated latent space, and the decoder reconstructed the corresponding 2,000 highly variable genes in scRNA from the cell embeddings. Accordingly, the model proved to be especially powerful in that it could predict the expression of genes in scRNA data, even though not measured in MERFISH data, in an online manner.

To assess the validity and qualification of online imputed scRNA data, we calculated the correlation of mean gene expression of real and predicted scRNA data in different cell types (Supplementary Fig. 6a). As a result, real and predicted scRNA data were significantly correlated, according to the Pearson correlation coefficient $R^2 = 0.999$ for all data, and ranged from 0.501 for Ependymal cells to 0.990 for Inhibitory cells. Results showed that the high-expression regions of common genes in MERFISH and generated scRNA were consistent in the latent space. Finally, for verification of cell-type labels, we plotted the top differential marker gene expression for each cell type in predicted scRNA data and observed consistent patterns of cell-type-specific expression (Supplementary Fig. 6b). For example, Excitatory and Inhibitory cells are difficult to distinguish, since, in essence, they belong to neural subtypes. However, our generated differential marker gene expression exhibited significant differences between the two cell types, such as marker genes Ghrh and Trh in Excitatory cells and Gal and Th in Inhibitory cells. We further demonstrated the high Pearson correlation coefficient between predicted marker genes and real MERFISH marker genes

(Supplementary Fig. 6c).

Supplementary Discussion 2: uniPort integrates datasets without dataset-specific genes

We introduced a uniPort version that only used common genes for integration, named uniPort-cm for convenience. Compared with uniPort-cm, uniPort leveraged common, ATAC- and RNA-specific genes for integration and demonstrated better integration performance (Supplementary Fig. 9). To intuitively show the importance of specific genes, we visualized the gene expression of RNA-specific marker genes GATA3 and MAF, and ATAC-specific marker genes JAKMIP1 and LINCO495 (Supplementary Fig. 10). These marker genes showed high expression values in CD4 and CD8 cells, which assisted in the identification and separation of corresponding cells.

Supplementary Discussion 3: Computational cost

We tested the scalability of uniPort to large-scale data by measuring both maximum memory usage and total runtime (Supplementary Fig. 12). To test uniPort's scalability against other methods, we sampled PBMC data (11,259 cells) to create seven benchmark datasets with $5K$, $10K$, $20K$, $40K$, $80K$, $160K$ and $320K$ cells. uniPort, SCALEX, scVI and Harmony scaled well beyond $320K$ cells, while MultiMAP, Seurat and LIGER is not suitable for integration of datasets beyond $160K$ cells in this case. In sum, the minibatch strategy in the deep learning framework dramatically reduced uniPort's time and memory consumption. For example, uniPort required only 19.4 gigabytes (GB) for $320K$ cells, better than other methods except for Harmony. Although uniPort consumed more runtime when cell number is less than $20K$ cells, which was about twice that of SCALEX owing to the addition of optimal transport computation, it was still very efficient and consumed almost constant runtime (about 11 minutes) with the cell number increased.

We further plotted the computational time of more compared methods on the PBMC example (Supplementary Fig. 11). The result demonstrated that uniPort had competitive efficiency with other VAE-based models, and took much less time than global optimal transport methods, such as SCOT and Pamona.

Supplementary Discussion 4: uniPort integrates datasets profiled from the same cells

We also developed a method in uniPort for integrative clustering of multiple datasets simultaneously profiled from the same cells (set *mode='v'* in *uniport.Run* function in *uniport* Python package). This aims to improve the clustering performance of one modality with the information of other modalities. It takes one dataset as input and projects the data into a latent space using an encoder. Then, uniPort reconstructs different modalities through different decoders, which revises the clustering performance in the latent space. We tested the clustering performance of uniPort with paired-cell CITE-seq [7] (Supplementary Fig. 14) and SNARE-seq data [6] (Supplementary Fig. 15).

We used CITE-seq data to help improve the clustering performance of scRNA data. Results showed that without the integration of CITE data, the Silhouette score of scRNA data in the latent space through encoder was 0.621 (Supplementary Fig. 14b). However, if we involved the information of CITE-seq data, the Silhouette score increased to 0.680 (Supplementary Fig. 14c). We also tested the change of the Silhouette score with different balanced parameter λ_s, which reflects the importance of CITE data (Supplementary Fig. 14d). The curve showed that with the increase of λ_s, the Silhouette increased to 0.680 ($\lambda_s = 0.2$) first and then decreased. Therefore, we suggest the parameter be set from 0 to 0.5.

We also used scRNA to help improve the cluster peformance of scATAC peaks in SNARE-seq data [6]. Results showed that without the integration of scRNA data, scATAC demonstrated completely chaotic in the latent space (Supplementary Fig. 15b). However, with the information of scRNA, different scATAC clusters showed obvious separation (Supplementary Fig. 15c).

Supplementary Discussion 5: uniPort integrates datasets without aligned common features

uniPort framework also involves a method for integration of datasets without common features (set *mode='d'* in *uniport.Run* function in *uniport* Python package). uniPort takes two single-cell datasets with distinct features as input, which means there is no common gene as reference

for alignment (Supplementary Fig. 16a). We employ two dataset-specific encoders ψ_x and ψ_y to project the datasets into the same dimensional cell-embedding latent space and perform Minibatch-UOT to align the cells. Afterwards, two dataset-specific decoders ϕ_x and ϕ_y are used to reconstruct the inputs, respectively.

We tested the performance of uniPort with scRNA data and original scATAC peaks data instead of gene activity matrix in PBMC datasets, which means the two modalities share no common features. The results showed that uniPort successfully integrated some cell types, including CD4 Naive and CD14 Mono cells, but failed to integrate other cell types (Supplementary Fig. 16b), which is reasonable owing to the lack of reference information, neither from cells nor genes.

Supplementary Discussion 6. uniPort is robust to the number of selected common and dataset-specific HVGs.

we evaluated the robustness of uniPort on the number of common and data-specific HVG selected on the paired PBMC example with three scenarios as follows: 1) We selected 8000, 4000, 2000, 1000 and 500 genes, respectively, for both common and dataset-specific HVGs for integration; 2) We selected 8000, 4000, 2000, 1000 and 500 genes, respectively, for dataset-specific HVGs, while maintained number of common HVGs as 2000 for integration; 3) We selected 8000, 4000, 2000, 1000 and 500 genes, respectively, for common HVGs, while maintained number of common HVGs as 2000 for integration. The result showed that uniPort was robust to different choices of the number of common and data-specific HVGs (Supplementary Fig. 17b).

Supplementary Figures

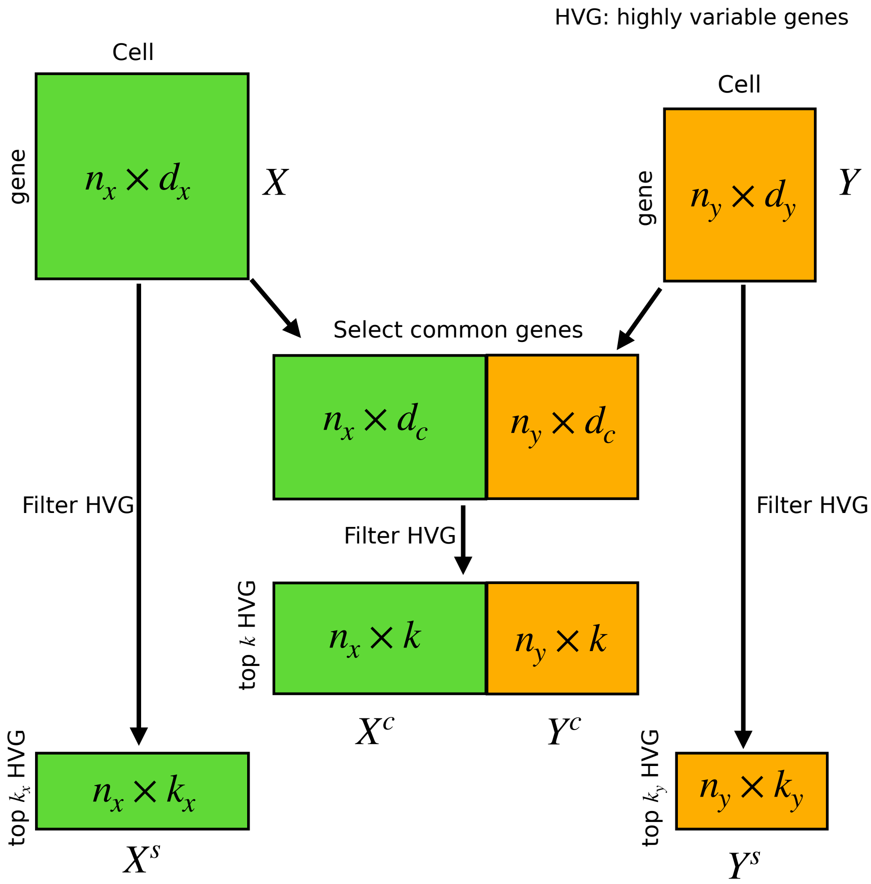

Supplementary Figure 1. Gene selection steps. Steps for selecting k common highly variable genes (HVG), and k_x dataset-specific HVG of dataset $\mathbf{X}$ and k_y dataset-specific HVG of dataset $\mathbf{Y}$.

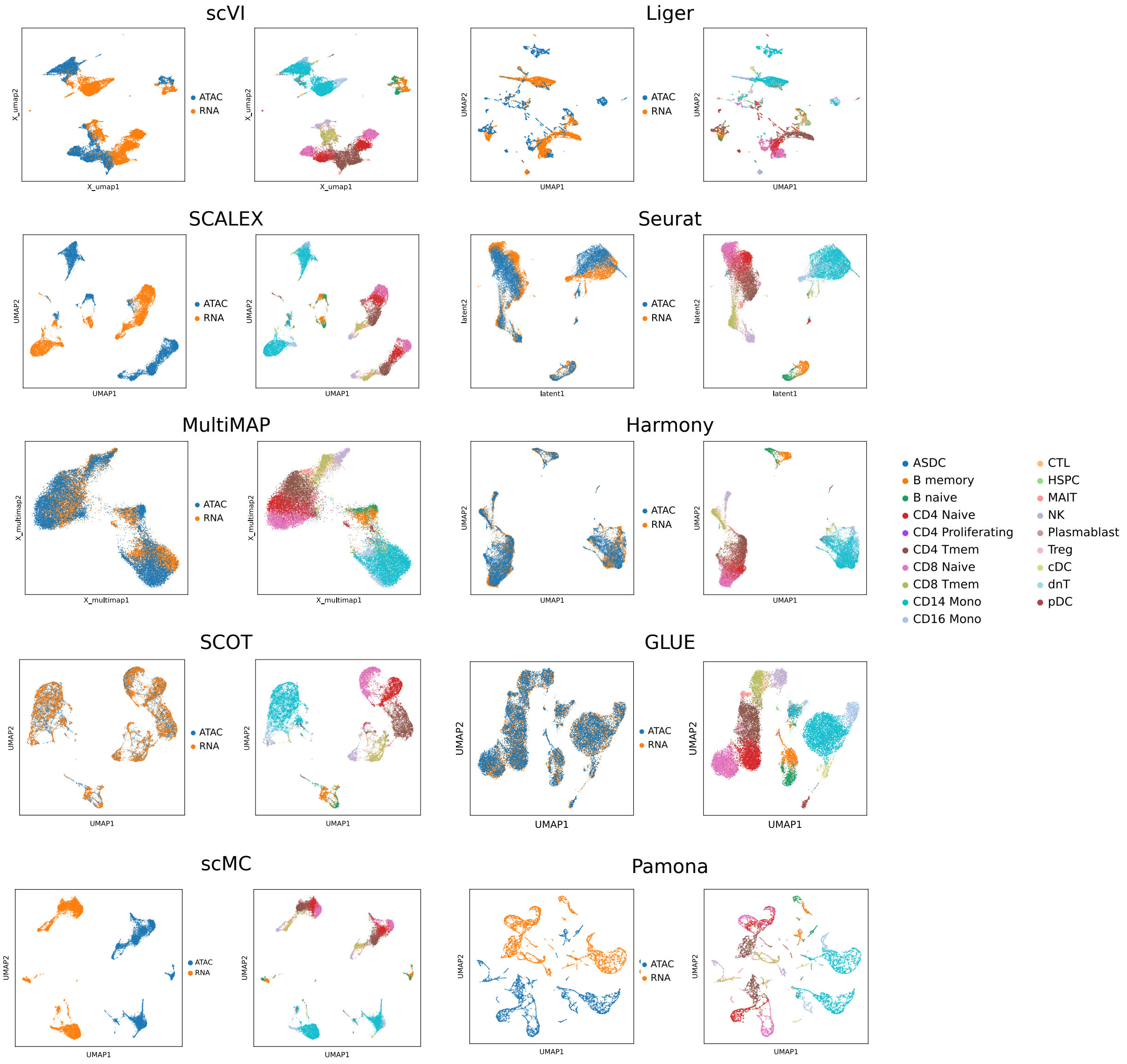

Supplementary Figure 2. UMAP visualization of different methods on the paired PBMC example.

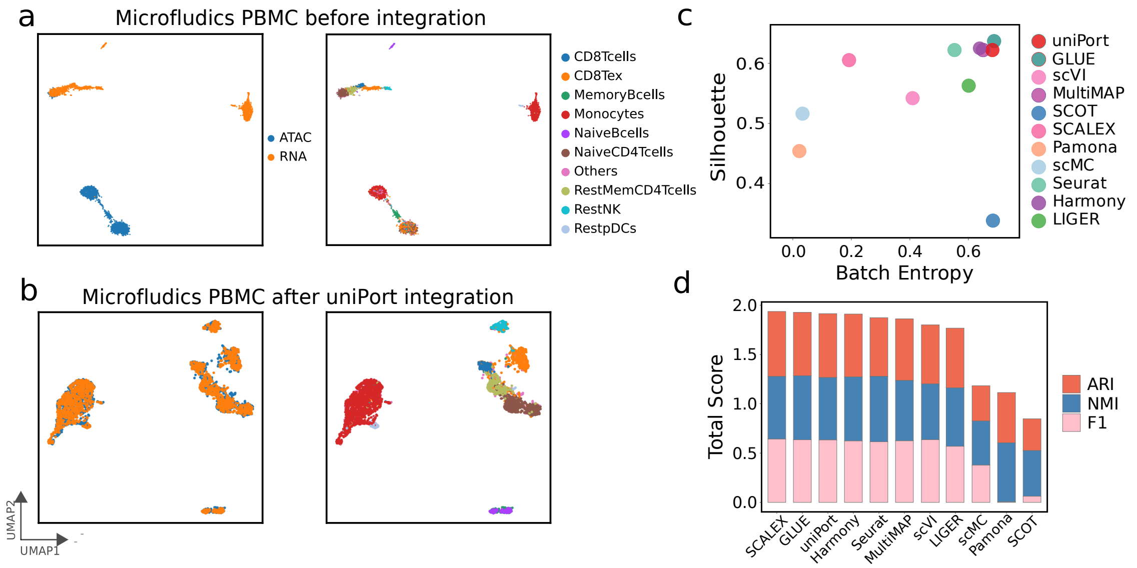

Supplementary Figure 3. uniPort integrates the unpaired Microfludic-based PBMC dataset. a, UMAP visualization of Microfludics PBMC before integration. **b,** UMAP visualization of Microfludics PBMC after uniPort integration. **c,** Comparison of Batch Entropy scores and Silhouette coefficients of different methods. **d,** Comparison of total scores of ARI, NMI and F1 of different methods.

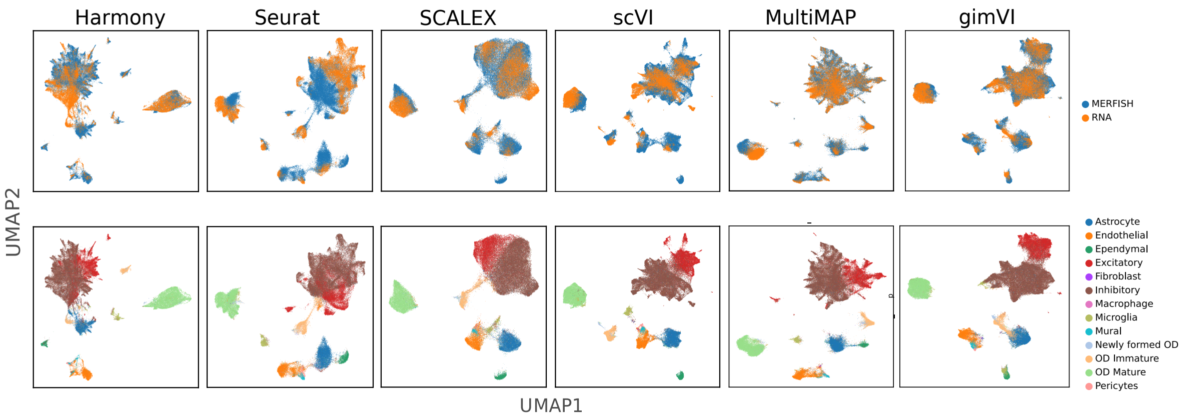

Supplementary Figure 4. UMAP visualization of integration results of compared methods on the MERFISH example.

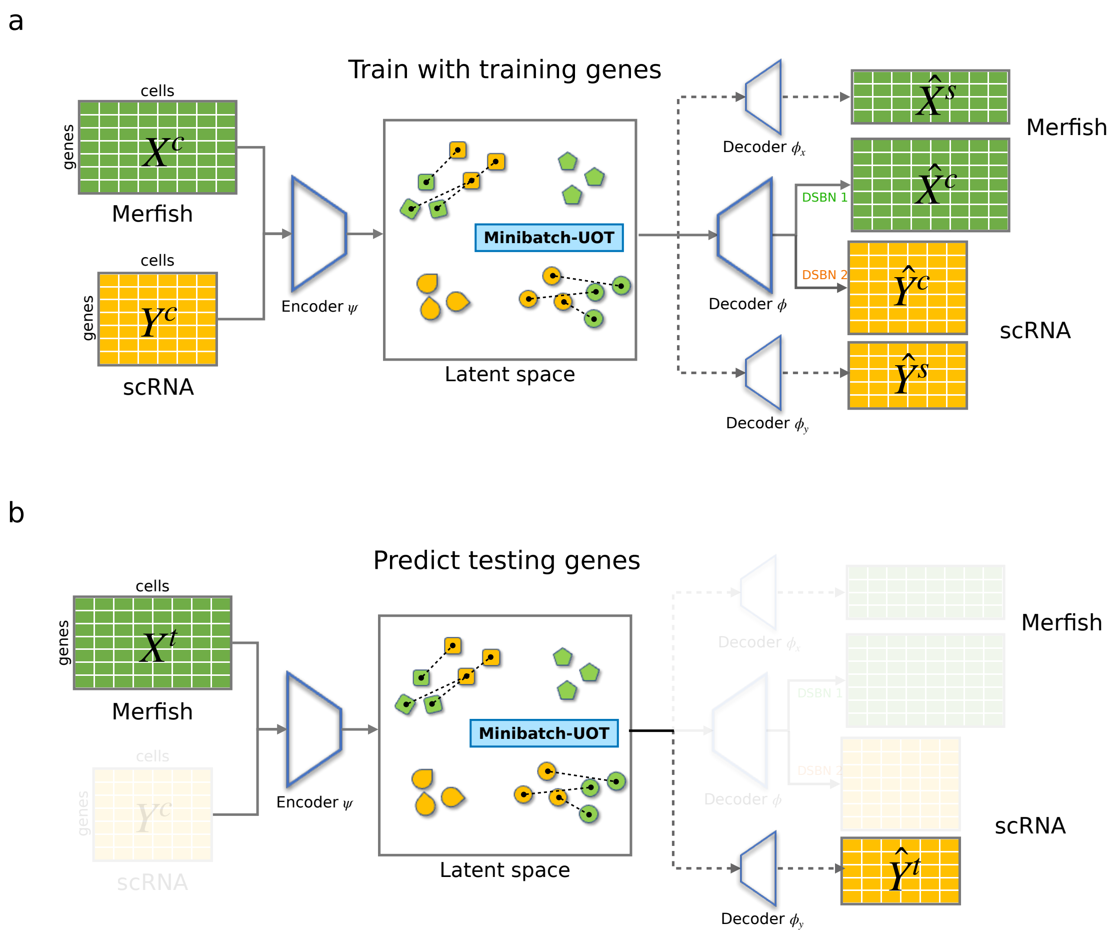

Supplementary Figure 5. uniPort imputes MERFISH genes. a, uniPort trains an atlas on the scRNA and MERFISH data. **b,** uniPort imputes scRNA-specific genes through MERFISH input.

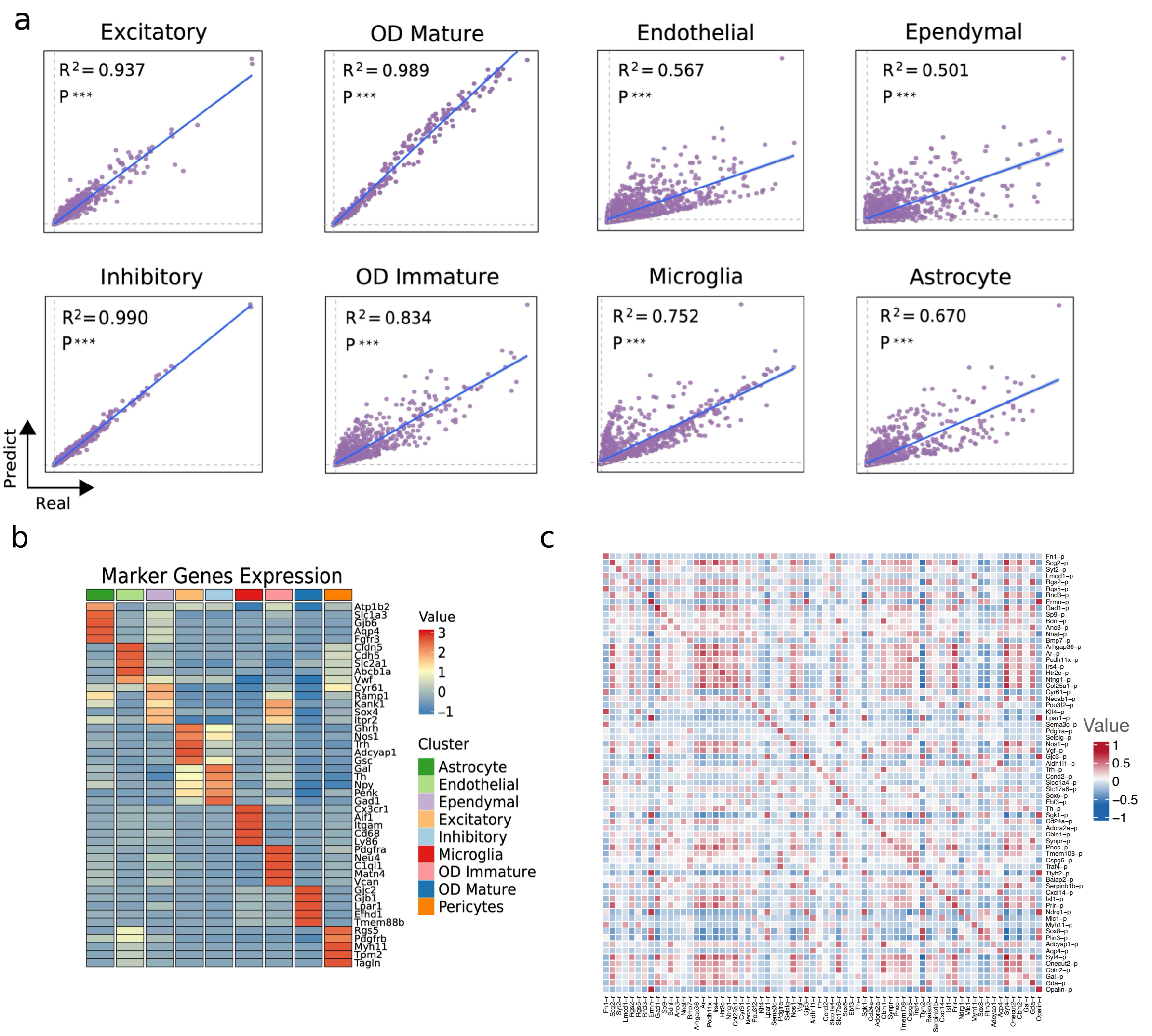

Supplementary Figure 6. Online gene imputation for mouse #2. a, Correlation of mean predicted and real expression of RNA genes. **b**, Mean marker gene expression in different cell types. **c**, Pearson correlation coefficient between imputed marker genes and real MERFISH marker genes of mouse #2 (two-sided t-test, p*** < 0.001.).

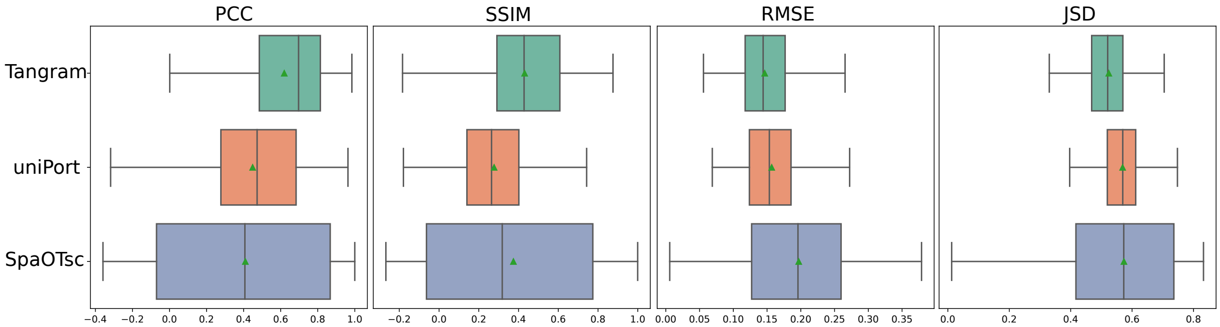

Supplementary Figure 7. Spatial deconvolution benchmark. Pearson correlation coefficient (PCC), structural similarity index (SSIM), root mean square error (RMSE) and Jensen-Shannon divergence (JSD) of uniPort, Tangram and SpaOTsc on the simulated STARmap example (n=189). Higher PCC and SSIM and lower RMSE and JSD, indicate better performance. In the boxplot, the center line, box limits and whiskers denote the median, upper and lower quartiles and 1.5× interquartile range, respectively.

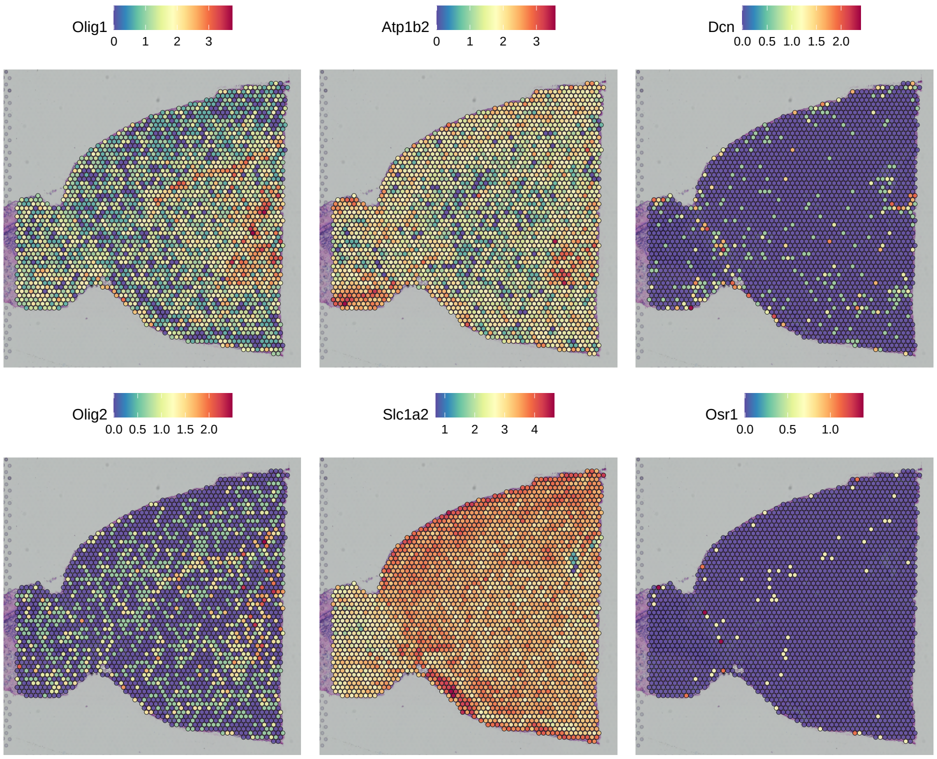

Supplementary Figure 8. Marker genes in adult mouse brain ST data. Left panel: Olig1 and Olig2 genes of cluster Oligo; Middle panel: Atp1b2 and Slc1a2 genes of cluster Astro; Right panel: Dcn and Osr1 genes of cluster VLMC.

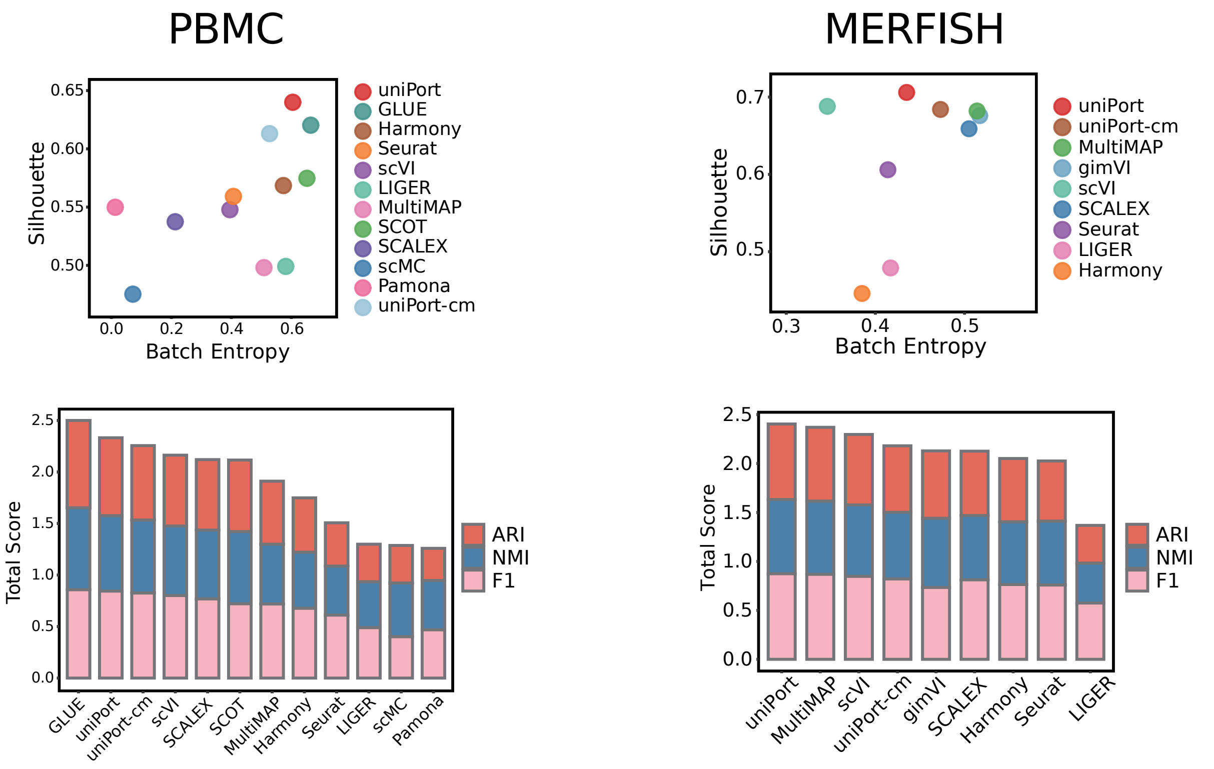

Supplementary Figure 9. Integration peformance of uniPort on PBMC and MERFISH examples without dataset-specific genes.

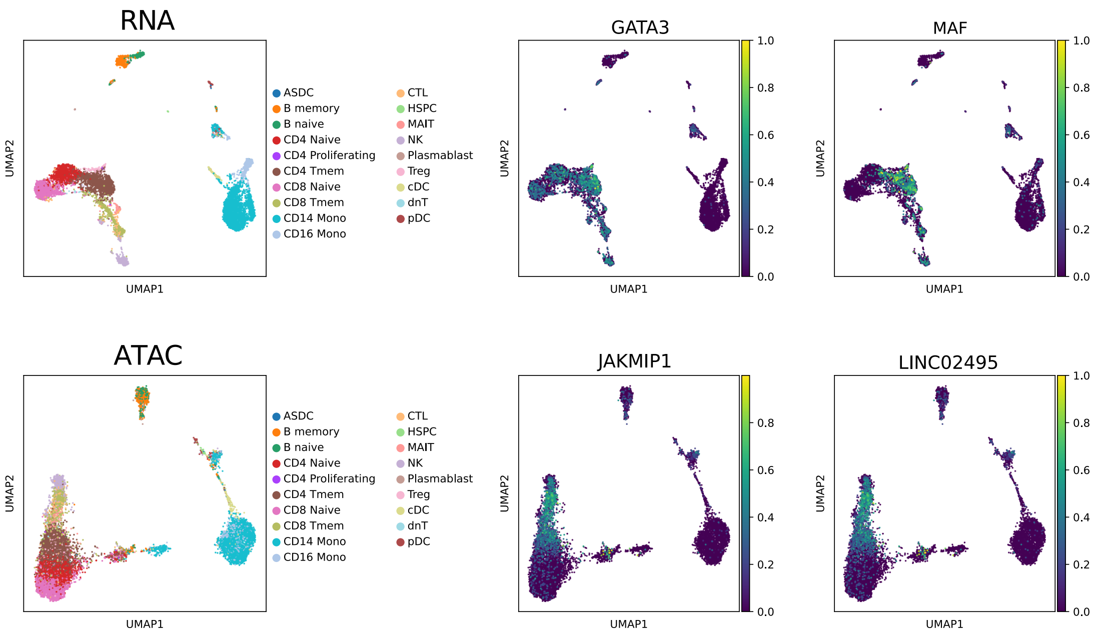

Supplementary Figure 10. Examples of RNA and ATAC Specific genes for uniPort integration.

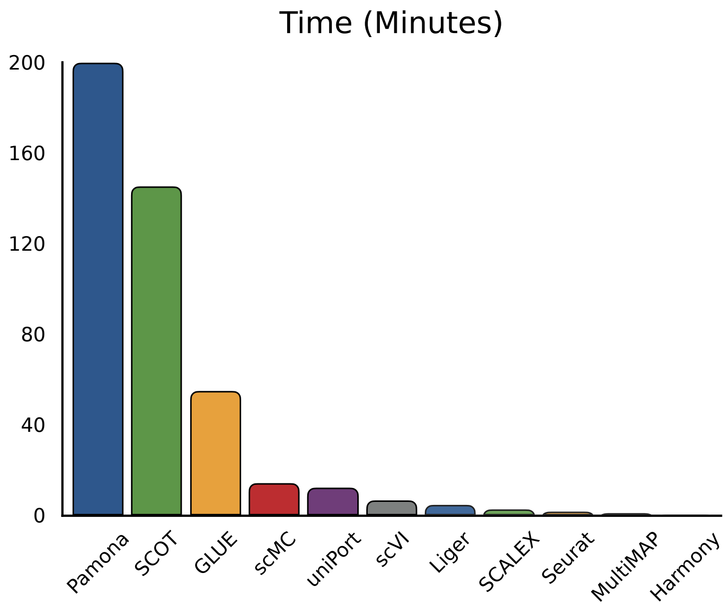

Supplementary Figure 11. Running time of different methods on PBMC example.

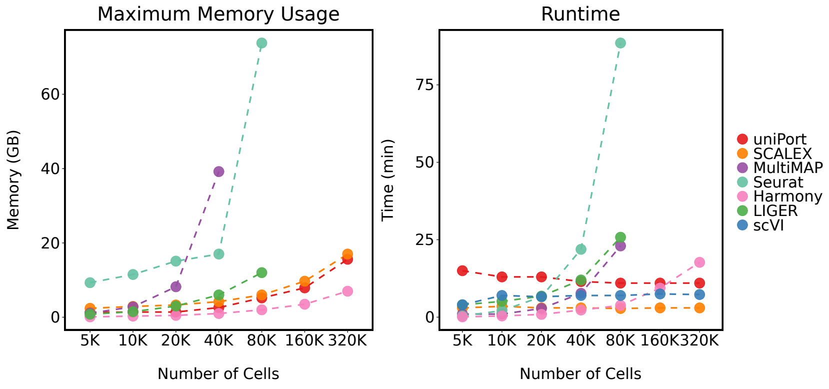

Supplementary Figure 12. Computational cost. Maximum memory usage and total runtime of part of compared methods with different data sizes.

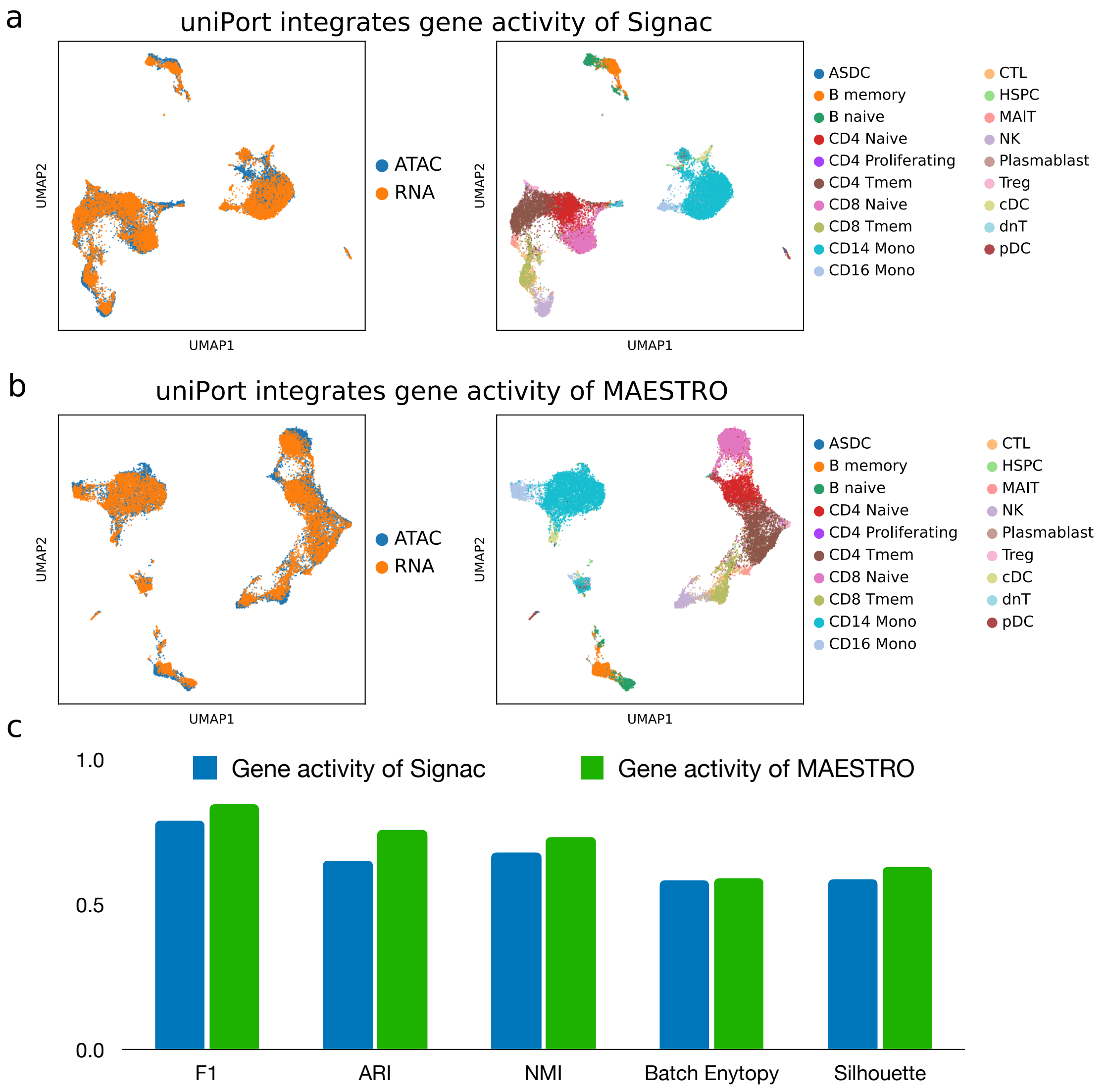

Supplementary Figure 13. Comparison of uniPort performance on different gene activity scores. a, UMAP visualization of uniPort PBMC integration on gene activity of Signac. **b,** UMAP visualization of uniPort PBMC integration by gene activity of MAESTRO. **c,** Comparison of F1, ARI, NMI, Batch Entropy scores and Silhouette coefficients by gene activity of Signac and MAESTRO.

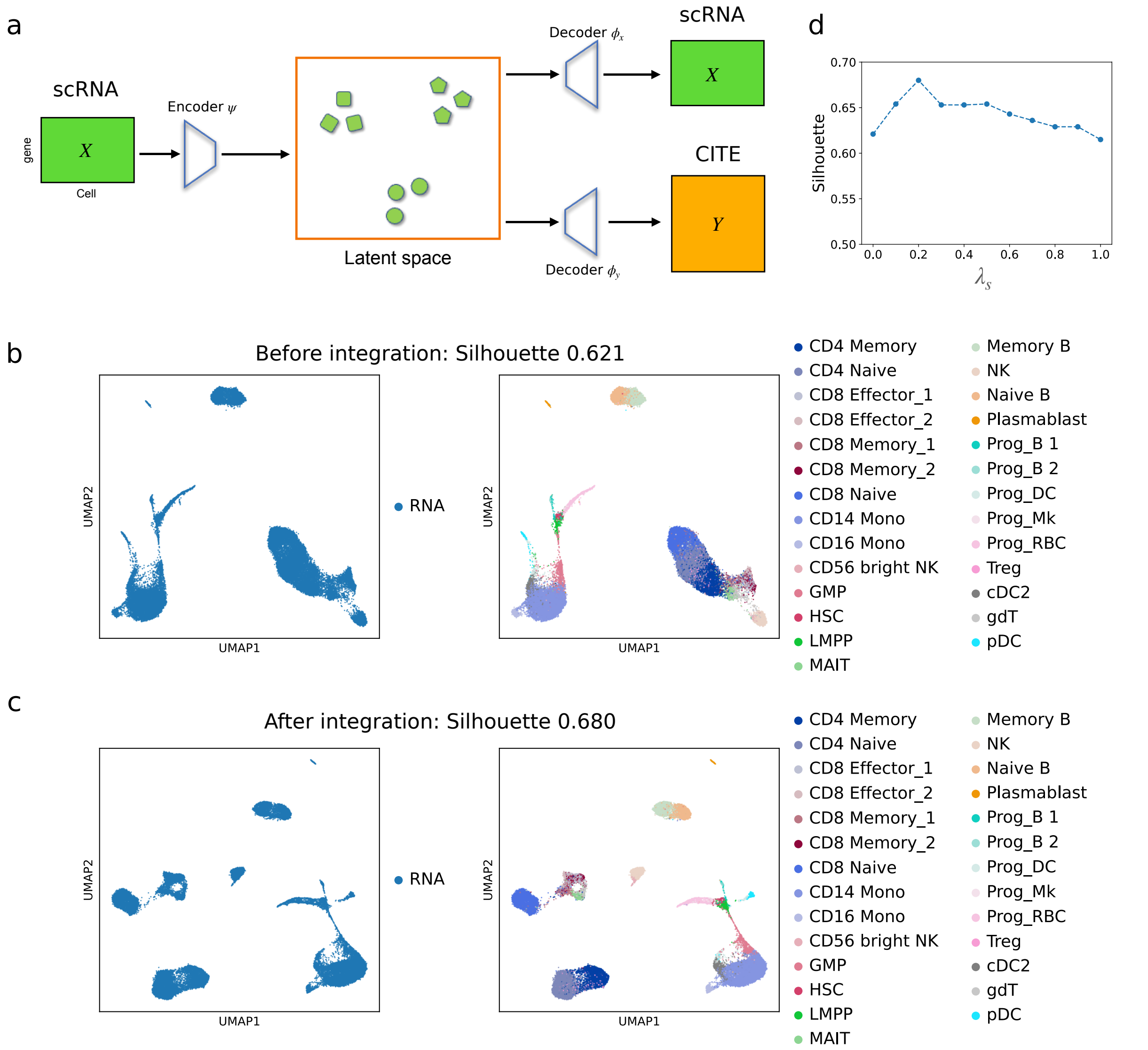

Supplementary Figure 14. Vertical integration. **a**, uniPort takes scRNA data as input and projects it into a low-dimensional latent space by a probabilistic decoder. Then uniPort reconstructs both scRNA and CITE-seq data through their specific decoders from cell embeddings in the latent space. **b**, UMAP visualization of scRNA data without integration of CITE data. **c**, UMAP visualization of scRNA data with integration of CITE data. **d**, The change of Silhouette score with different choices of λ_s parameter.

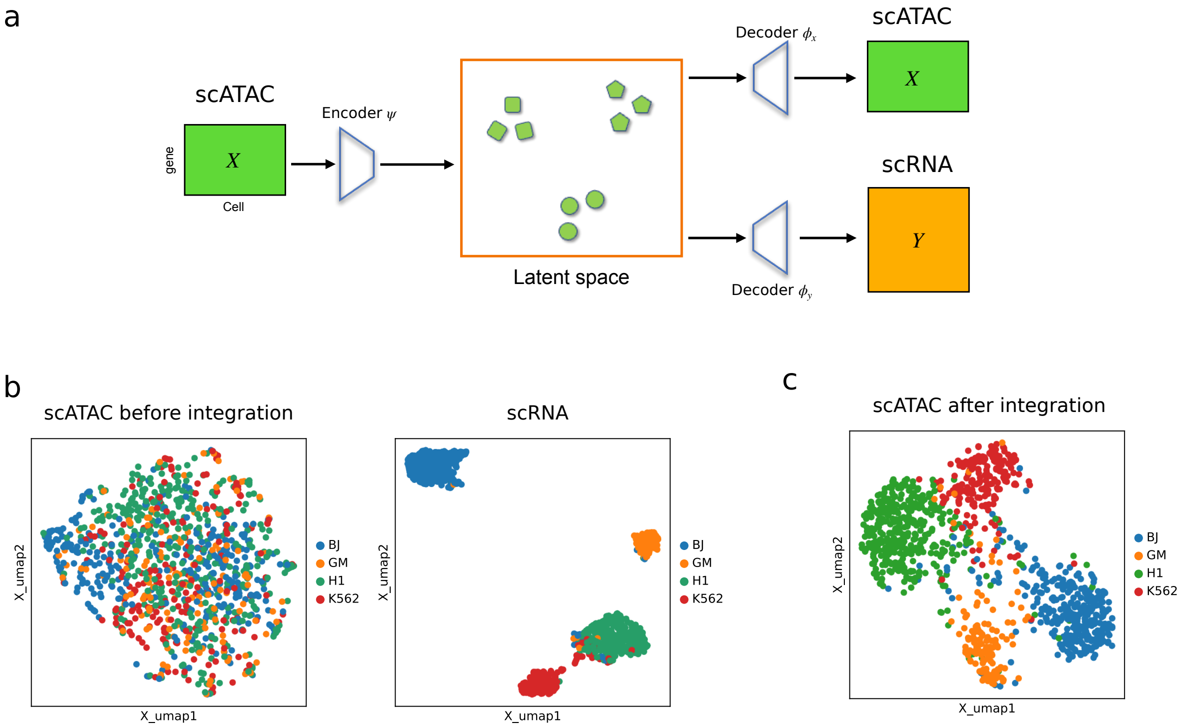

Supplementary Figure 15. Vertical integration for SNARE data. a, uniPort takes scATAC data as input and projects it into a low-dimensional latent space by a probabilistic decoder. Then uniPort reconstructs both scATAC and scRNA data through their specific decoders from cell embeddings in the latent space. **b**, UMAP visualization of scATAC data without integration of scRNA data. **c**, UMAP visualization of scATAC data with integration of scRNA data.

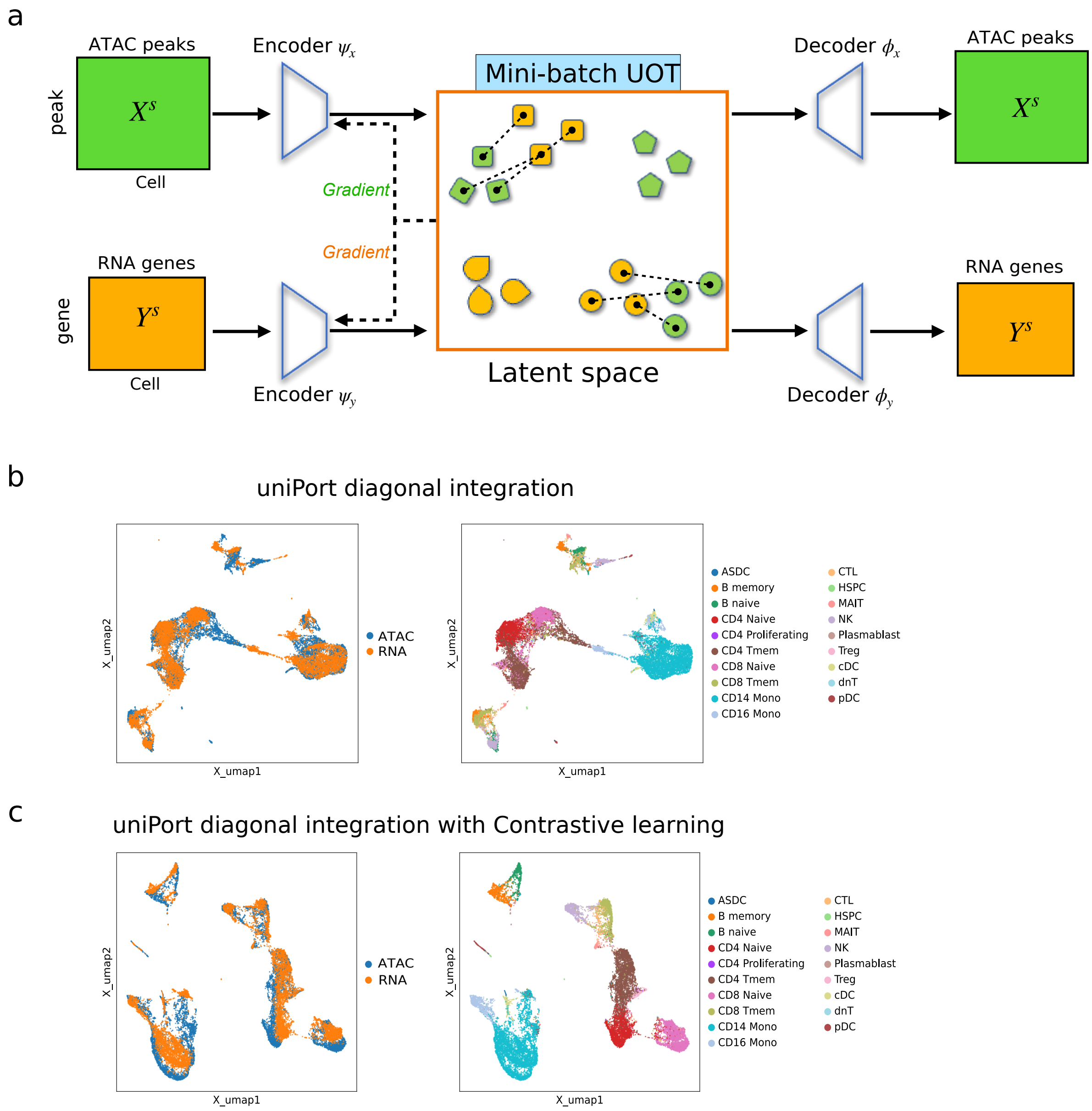

Supplementary Figure 16. Diagonal integration. **a**, uniPort takes scATAC peaks and scRNA without aligned common genes as input, and projects them into a common cell-embedding space by different encoders. Then uniPort minimizes the Minibatch-UOT loss and reconstructs input through corresponding decoders. **b**, UMAP visualization of integration result of PBMC by uniPort. **c**, UMAP visualization of PBMC data by uniPort with contrastive learning.

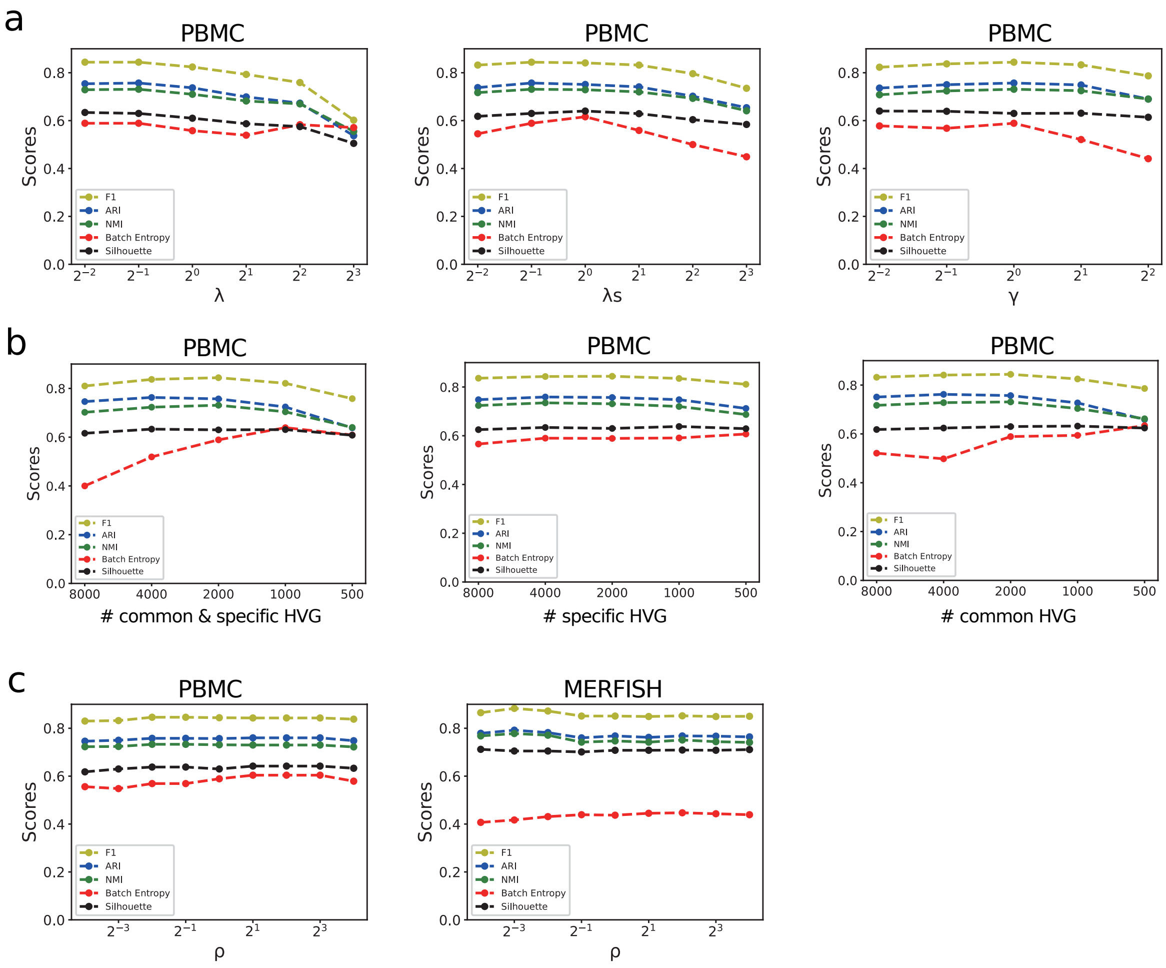

Supplementary Figure 17. The robustness of uniPort corresponding to different choices of parameters over the paired PBMC example. **a**, Evaluated scores of uniPort for different choices of λ, λ_s and γ over the paired PBMC example. **b**, Evaluated scores of uniPort when selecting different numbers of common and/or specific HVG over the paired PBMC example. **c**, Evaluated scores of uniPort for different choices of ρ over the paired PBMC and MERFISH examples.

Supplementary References

[1] Jean-David Benamou, Guillaume Carlier, Marco Cuturi, Luca Nenna, and Gabriel Peyré. Iterative Bregman Projections for Regularized Transportation Problems. *SIAM Journal on Scientific Computing*, 2(37):A1111–A1138, 2015.

[2] Kai Cao, Yiguang Hong, and Lin Wan. Manifold alignment for heterogeneous single-cell multi-omics data integration using Pamona. *Bioinformatics*, 08 2021.

[3] Nicolas Courty, Rémi Flamary, Amaury Habrard, and Alain Rakotomamonjy. Joint distribution optimal transportation for domain adaptation. In *Proceedings of the 31st International Conference on Neural Information Processing Systems*, NIPS'17, page 3733–3742, Red Hook, NY, USA, 2017. Curran Associates Inc.

[4] Kilian Fatras, Thibault Séjourné, Rémi Flamary, and Nicolas Courty. Unbalanced minibatch optimal transport; applications to domain adaptation. In *International Conference on Machine Learning*, pages 3186–3197. PMLR, 2021.

[5] Elad Hoffer and Nir Ailon. Deep metric learning using triplet network. In Aasa Feragen, Marcello Pelillo, and Marco Loog, editors, *Similarity-Based Pattern Recognition*, pages 84–92, Cham, 2015. Springer International Publishing.

[6] Isac Lee, Roham Razaghi, Timothy Gilpatrick, Michael Molnar, Ariel Gershman, Norah Sadowski, Fritz J. Sedlazeck, Kasper D. Hansen, Jared T. Simpson, and Winston Timp. Simultaneous profiling of chromatin accessibility and methylation on human cell lines with nanopore sequencing. *Nature Methods*, 17(12):1191–1199, Dec 2020.

[7] Marlon Stoeckius, Christoph Hafemeister, William Stephenson, Brian Houck-Loomis, Pratip K Chattopadhyay, Harold Swerdlow, Rahul Satija, and Peter Smibert. Simultaneous epitope and transcriptome measurement in single cells. *Nature Methods*, 14(9):865–868, 2017.

[8] Asuka Takatsu. On wasserstein geometry of gaussian measures. In *Probabilistic approach to geometry*, pages 463–472. Mathematical Society of Japan, 2010.

[9] Yujia Xie, Xiangfeng Wang, Ruijia Wang, and Hongyuan Zha. A fast proximal point method for computing exact wasserstein distance. In *Uncertainty in Artificial Intelligence*, pages 433–453. PMLR, 2020.

[10] Hongteng Xu, Dixin Luo, Ricardo Henao, Svati Shah, and Lawrence Carin. Learning autoencoders with relational regularization. In *International Conference on Machine Learning*, pages 10576–10586. PMLR, 2020.

[11] Jinglong Zhang, Xu Zhang, Ying Wang, Feng Zeng, and Xing-Ming Zhao. MAT2: manifold alignment of single-cell transcriptomes with cell triplets. *Bioinformatics*, 37(19):3263–3269, 05 2021.
