# Supplementary material for: A unified computational framework for single-cell data integration with optimal transport
Source: Nat Commun. 2022 Dec 1;13:7419. doi: 10.1038/s41467-022-35094-8 (PMC9715710; doi:10.1038/s41467-022-35094-8)
Supplement: Supplementary file 3 — Description of additional Supplementary File [file 41467_2022_35094_MOESM3_ESM.pdf]

Description of additional supplementary Files

Supplementary Data 1 : links to all data sources.
