# Supplementary material for: A unified computational framework for single-cell data integration with optimal transport
Source: Nat Commun. 2022 Dec 1;13:7419. doi: 10.1038/s41467-022-35094-8 (PMC9715710; doi:10.1038/s41467-022-35094-8)
Supplement: Supplementary file 5 — Reporting Summary [file 41467_2022_35094_MOESM5_ESM.pdf]

nature portfolio

Corresponding author(s): Yiguang Hong, Lin Wan

Last updated by author(s): Nov 8, 2022

Reporting Summary

Nature Portfolio wishes to improve the reproducibility of the work that we publish. This form provides structure for consistency and transparency in reporting. For further information on Nature Portfolio policies, see our Editorial Policies and the Editorial Policy Checklist.

Statistics

For all statistical analyses, confirm that the following items are present in the figure legend, table legend, main text, or Methods section.

n/a	Confirmed	
☐	☒	The exact sample size (n) for each experimental group/condition, given as a discrete number and unit of measurement
☒	☐	A statement on whether measurements were taken from distinct samples or whether the same sample was measured repeatedly
☐	☒	The statistical test(s) used AND whether they are one- or two-sided<br>*Only common tests should be described solely by name; describe more complex techniques in the Methods section.*
☒	☐	A description of all covariates tested
☒	☐	A description of any assumptions or corrections, such as tests of normality and adjustment for multiple comparisons
☐	☒	A full description of the statistical parameters including central tendency (e.g. means) or other basic estimates (e.g. regression coefficient) AND variation (e.g. standard deviation) or associated estimates of uncertainty (e.g. confidence intervals)
☐	☒	For null hypothesis testing, the test statistic (e.g. F, t, r) with confidence intervals, effect sizes, degrees of freedom and P value noted<br>*Give P values as exact values whenever suitable.*
☒	☐	For Bayesian analysis, information on the choice of priors and Markov chain Monte Carlo settings
☒	☐	For hierarchical and complex designs, identification of the appropriate level for tests and full reporting of outcomes
☐	☒	Estimates of effect sizes (e.g. Cohen's d, Pearson's r), indicating how they were calculated

Our web collection on statistics for biologists contains articles on many of the points above.

Software and code

Policy information about availability of computer code

Data collection	No software was used for data collection.
Data analysis	The software of uniPort is downloadable from pip and is available at https://github.com/caokai1073/uniPort. The data preprocessing and analysis of results were done using R 4.0.2 and package Seurat v4.1.0, Signac v1.5.0, Python 3.8.13 and package Scanpy v1.8.2.<br>Other software used in this paper: Seurat (v4.1.0), LIGER (v1.0.0), Harmony (v1.0), SCALEX (v0.2.0), MultiMAP (v0.0.1), scVI (v0.17.1), scMC (v1.0.0), GLUE (v0.2.3), SCOT (v1.0), Pamona (v0.1.0), Tangram (v1.0.3).

For manuscripts utilizing custom algorithms or software that are central to the research but not yet described in published literature, software must be made available to editors and reviewers. We strongly encourage code deposition in a community repository (e.g. GitHub). See the Nature Portfolio guidelines for submitting code & software for further information.

Data

Policy information about availability of data

All manuscripts must include a data availability statement. This statement should provide the following information, where applicable:

- Accession codes, unique identifiers, or web links for publicly available datasets
- A description of any restrictions on data availability
- For clinical datasets or third party data, please ensure that the statement adheres to our policy

All data analyzed in this article are publicly available through online sources. We present links to all data sources in Supplementary Data 1.

1. The paired PBMC data are available at https://www.10xgenomics.com/cn/resources/datasets/pbmc-from-a-healthy-donor-granulocytes-removed-through-cell-sorting-10-k-1-standard-2-0-0.
2. The microfluidics PBMC data are available at https://github.com/liulab-dfci/MAESTRO/tree/master/data.
3. The mouse spleen data are available at https://www.ebi.ac.uk/arrayexpress/experiments/E-MTAB-6714.
4. The MERFISH data are available at https://datadryad.org/stash/dataset/doi:10.5061/dryad.8t8s248. The matched scRNA data are available at GSE113576.
5. The synthetic STARmap data are available at https://github.com/QuKunLab/SpatialBenchmarking/tree/main/FigureData/Figure4/Dataset10_STARmap/Simulated_STARmap.
5. The spatial data (10x Visium) of mouse brain are available at https://satijalab.org/seurat/articles/spatial_vignette.html. The matched scRNA data are available at https://marcelosua.github.io.
6. The spatial data (10x Visium) of breast cancer are available at https://www.10xgenomics.com/cn/resources/datasets/human-breast-cancer-ductal-carcinoma-in-situ-invasive-carcinoma-ffpe-1-standard-1-3-0. The matched scRNA data are available at https://singlecell.broadinstitute.org/single_cell/study/SCP1039/a-single-cell-and-spatially-resolved-atlas-of-human-breast-cancers#study-download.
8. The spatial and matched scRNA data of PDAC are available at GSE111672.
9. The PBMC CITE-seq data are available at https://satijalab.org/seurat.
10. The SNARE-seq data are available at GSE126074.

Human research participants

Policy information about studies involving human research participants and Sex and Gender in Research.

Reporting on sex and gender	None.
Population characteristics	None.
Recruitment	None.
Ethics oversight	None.

Note that full information on the approval of the study protocol must also be provided in the manuscript.

Field-specific reporting

Please select the one below that is the best fit for your research. If you are not sure, read the appropriate sections before making your selection.

[x] Life sciences   [ ] Behavioural & social sciences   [ ] Ecological, evolutionary & environmental sciences

For a reference copy of the document with all sections, see nature.com/documents/nr-reporting-summary-flat.pdf

Life sciences study design

All studies must disclose on these points even when the disclosure is negative.

Sample size	We applied uniPort to eight datasets, including three scATAC and scRNA datasets, one high-plex RNA imaging-based MERFISH dataset, and four barcoding-based spatial datasets. Therefore, it is sufficient to demonstrate the functionalities of uniPort.
Data exclusions	Standard filtering procedures were performed to exclude low-quality cells and low-count genes.
Replication	We independently tested on benchmarking datasets. All results were replicable by running our algorithm.
Randomization	Complete randomization was performed for allocating groups.
Blinding	Our study does not involve group allocation that requires blinding.

Reporting for specific materials, systems and methods

We require information from authors about some types of materials, experimental systems and methods used in many studies. Here, indicate whether each material, system or method listed is relevant to your study. If you are not sure if a list item applies to your research, read the appropriate section before selecting a response.

Materials & experimental systems

n/a	Involved in the study
☒	☐ Antibodies
☒	☐ Eukaryotic cell lines
☒	☐ Palaeontology and archaeology
☒	☐ Animals and other organisms
☒	☐ Clinical data
☒	☐ Dual use research of concern

Methods

n/a	Involved in the study
☒	☐ ChIP-seq
☒	☐ Flow cytometry
☒	☐ MRI-based neuroimaging
